# A graph modification approach for finding core–periphery structures in protein interaction networks

**DOI:** 10.1186/s13015-015-0043-7

**Published:** 2015-05-02

**Authors:** Sharon Bruckner, Falk Hüffner, Christian Komusiewicz

**Affiliations:** International Max Planck Research School for Computational Biology and Scientific Computing, Ihnestr. 63-73, Berlin, 14195 Germany; Institut für Softwaretechnik und Theoretische Informatik, TU Berlin, Ernst-Reuter-Platz 7, Berlin, 10587 Germany

**Keywords:** Protein complexes, Graph classes, NP-hard problems

## Abstract

The core–periphery model for protein interaction (PPI) networks assumes that protein complexes in these networks consist of a dense core and a possibly sparse periphery that is adjacent to vertices in the core of the complex. In this work, we aim at uncovering a global core–periphery structure for a given PPI network. We propose two exact graph-theoretic formulations for this task, which aim to fit the input network to a hypothetical ground truth network by a minimum number of edge modifications. In one model each cluster has its own periphery, and in the other the periphery is shared. We first analyze both models from a theoretical point of view, showing their NP-hardness. Then, we devise efficient exact and heuristic algorithms for both models and finally perform an evaluation on subnetworks of the *S. cerevisiae* PPI network.

**Electronic supplementary material:**

The online version of this article (doi:10.1186/s13015-015-0043-7) contains supplementary material, which is available to authorized users.

## Background

A fundamental task in the analysis of PPI networks is the identification of protein complexes and functional modules. Herein, a basic assumption is that complexes in a PPI network are strongly connected among themselves and weakly connected to other complexes [1]. This assumption is usually too strict. To obtain a more realistic network model of protein complexes, several approaches incorporate the *core–attachment* model of protein complexes [2]. In this model, a complex is conjectured to consist of a stable core plus some attachment proteins, which have only transient interactions with the core. In graph-theoretic terms, the core thus is a dense subnetwork of the PPI network. The attachment (or: periphery) is less dense, but has edges to one or more cores.

Current methods employing this type of modeling are based on *seed growing* [3-5]. Here, an initial set of promising small subgraphs is chosen as cores. Then, each core is separately greedily expanded by adding vertices to its core or its attachment (in each step, a vertex maximizing some specific objective function is chosen). The aim of these approaches was to predict protein complexes [4,5] or to reveal biological features that are correlated with topological properties of core–periphery structures in networks [3]. In this work, we use core–periphery modeling in a different context. Instead of searching for *local* core–periphery structures, we attempt to unravel a *global* core–periphery structure in PPI networks.

To this end, we hypothesize that the true network consists of several core–periphery structures. We propose two precise models to describe this. In the first model, the core–periphery structures are disjoint. In the second model, the peripheries may interact with different cores, but the cores are disjoint. Then, we fit the input data to each formal model and evaluate the results on several PPI networks.

**Our approach.** In spirit, our approach is related to the clique-corruption model of the CAST algorithm for gene expression data clustering [6]. In this model, the input is a similarity graph where edges between vertices indicate similarity. The hypothesis is that the objects corresponding to the vertices belong to disjoint biological groups of similar objects, the clusters. In the case of gene expression data, these are assumed to be groups of genes with the same function. Assuming perfect measurements, the similarity graph is a cluster graph.

### **Definition1**.

A graph *G* is a *cluster graph* if each connected component of *G* is a clique.

Because of stochastic measurement noise, the input graph is not a cluster graph. The task is to recover the underlying cluster graph from the input graph. Under the assumption that the errors are independent, the most likely cluster graph is one that disagrees with the input graph on a minimum number of edges. Such a graph can be found by applying a minimum number of edge modifications (that is, edge insertions or edge deletions) to the input graph. This paradigm directly leads to the optimization problem CLUSTER EDITING [7-9].

We now apply this approach to our hypothesis that there is a global core–periphery structure in the PPI networks. In both models detailed here, we assume that all proteins of each core interact with each other; this implies that each core is a clique. We also assume that the proteins in the periphery interact only with the cores but not with each other. Hence, the peripheries are independent sets.

In the first model, we assume that ideally the protein interactions give rise to *vertex-disjoint* core–periphery structures, that is, there are no interactions between different cores and no interactions between cores and peripheries of other cores. Then each connected component has at most one core which is a clique and at most one periphery which is an independent set. This is precisely the definition of a split graph.

### **Definition2**.

A graph *G*=(*V*,*E*) is a *split graph* if *V* can be partitioned into *V*_1_ and *V*_2_ such that *G*[ *V*_1_] is an independent set and *G*[ *V*_2_] is a clique.

We call the vertices in *V*_1_*periphery vertices* and the vertices in *V*_2_*core vertices*. Note that the partition for a split graph is not always unique. Split graphs have been previously used to model core–periphery structures in social networks [10]. There, however, the assumption is that the network contains exactly one core–periphery structure. We assume that each connected component is a split graph; we call graphs with this property *split cluster graphs*. Our fitting model is described by the following optimization problem.

SPLIT CLUSTER EDITING

**Input:** An undirected graph *G*=(*V*,*E*).

**Task:** Transform *G* into a split cluster graph by applying a minimum number of edge modifications.

In our second model, we allow the vertices in the periphery to be attached to an arbitrary number of cores, thereby connecting the cores. In this model, we thus assume that the cores are disjoint cliques and the vertices of the periphery are an independent set. Such graphs are called *monopolar* [11].

### **Definition3**.

A graph is *monopolar* if its vertex set can be two-partitioned into *V*_1_ and *V*_2_ such that *G*[ *V*_1_] is an independent set and *G*[ *V*_2_] is a cluster graph. The partition (*V*_1_,*V*_2_) is called *monopolar partition*.

Again, we call the vertices in *V*_1_ periphery vertices and the vertices in *V*_2_ core vertices. Our second fitting model now is the following.

MONOPOLAR EDITING

**Input:** An undirected graph *G*=(*V*,*E*).

**Task:** Transform *G* into a monopolar graph by applying a minimum number of edge modifications and output a monopolar partition.

**Figure 1:**
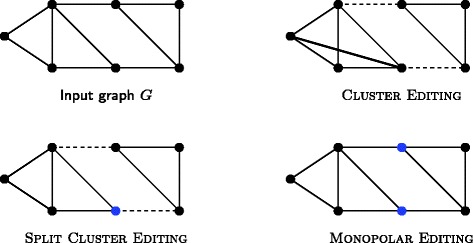
An example input and optimal solutions to CLUSTER EDITING, SPLIT CLUSTER EDITING, and MONOPOLAR EDITING. Dashed edges are edge deletions, bold edges are edge insertions. CLUSTER EDITING and SPLIT CLUSTER EDITING produce the same two clusters but SPLIT CLUSTER EDITING assigns the blue vertex of the size-four cluster to the periphery. In an optimal solution to MONOPOLAR EDITING the two blue vertices are in the periphery which is shared between two clusters. Note that the number of necessary edge modifications decreases from CLUSTER EDITING to SPLIT CLUSTER EDITING to MONOPOLAR EDITING.

Figure 1 shows an example graph along with optimal solutions for SPLIT CLUSTER EDITING and MONOPOLAR EDITING and, for comparison, CLUSTER EDITING. Clearly, the models behind SPLIT CLUSTER EDITING and MONOPOLAR EDITING are simplistic and cannot completely reflect biological reality. For example, subunits of protein complexes consisting of two proteins that first interact with each other and subsequently with the core of a protein complex are supported by neither of our models. Nevertheless, our models are less simplistic than pure clustering models that attempt to divide protein interaction networks into disjoint dense clusters. Furthermore, there is a clear trade-off between model complexity, algorithmic feasibility of models, and interpretability.

**Further related work.** In the following, we point to some related work in the literature that is not directly relevant for our algorithms and their evaluation but either considers models of core–periphery structure or optimization problems that are related to SPLIT CLUSTER EDITING or MONOPOLAR EDITING.

Della Rossa et al. [12] proposed to compute *core–periphery profiles* that assign to each vertex a numerical *coreness* value. The computation of these values is based on a heuristic random-walk model. Their evaluation showed that the *S. cerevisiae* PPI network exhibits a clear core–periphery structure which significantly deviates from random networks with the same degree distribution. An adaption of the Markov Clustering algorithm MCL that incorporates the core-attachment model for protein complexes was presented by Srihari et al. [13].

The SPLIT EDITING problem is closely related to SPLIT CLUSTER EDITING as it asks to transform a graph into a (single) split graph by at most *k* edge modifications. SPLIT EDITING is, somewhat surprisingly, solvable in linear time [14]; in fact, the number of required modifications depends only on the degree sequence. Thus, split graphs are recognizable by their degree sequence. Another problem that is related to CLUSTER EDITING is COGRAPH EDITING which asks to destroy induced *P*_4_’s by modifying at most *k* edges [15]. COGRAPH EDITING has applications in the computation of phylogenies [16]. In a cograph, every connected component has diameter at most two; in split cluster graphs every connected component has diameter at most three.

Finally, a further approach of fitting PPI networks to specific graph classes was proposed by Zotenko et al. [17] who find for a given PPI network a close chordal graph, that is, a graph without induced cycles of length four or more. The modification operation is insertion of edges. One notable difference is that the algorithm may be unable to construct a chordal graph from the input network [17].

**Preliminaries.** We consider undirected simple graphs *G*=(*V*,*E*) where *n*:=|*V*| denotes the number of vertices and *m*:=|*E*| denotes the number of edges. The *open neighborhood* of a vertex *u* is defined as *N*(*u*):={*v*∣{*u*,*v*}∈*E*}. We denote the *neighborhood of a setU* by $N(U):= \bigcup _{u\in U} N(u)\setminus U$. The *subgraph induced by a vertex setS* is defined as *G*[*S*]:=(*S*,{{*u*,*v*}∈*E*∣*u*,*v*∈*S*}).

One approach to solving NP-hard problems is based on the concept of fixed-parameter tractability [18,19]. Herein, instances *I* of a problem come along with a parameter *k*, for example the size of a solution. The aim is to obtain a fixed-parameter algorithm, that is, an algorithm with running time *f*(*k*)·*n*^*O*(1)^ where *f* depends only on *k*. Such an algorithm is efficient if *k* is small and *f* does not grow too rapidly.

The exponential-time hypothesis (ETH) states that there is a constant *c*>1 such that 3-SAT cannot be solved in (*c*−*ε*)^*n*^ time for any *ε*>0 [20]. Assuming the ETH, tight running time lower bounds can be shown; a survey on ETH-based running time lower bounds is given by Lokshtanov et al. [21]. If it is known that a parameterized problem *L* does not admit a 2^*o*(*k*)^·*n*^*O*(1)^-time algorithm (assuming the ETH), then a polynomial-time reduction from this problem to a problem *L*^′^ with parameter *k*^′^=*O*(*k*) implies that *L*^′^ cannot be solved in $\phantom {\dot {i}\!}2^{o(k^{\prime })}\cdot n^{O(1)}$ time (assuming the ETH). Note that the parameter in this case may also be the number of vertices or the number of edges of a graph.

## Combinatorial properties and complexity

Before presenting concrete algorithmic approaches for the two optimization problems, we show some properties of split cluster graphs and monopolar graphs which will be useful in the various algorithms. Furthermore, we present computational hardness results for the problems which will justify the use of integer linear programming (ILP) and heuristic approaches.

### Split cluster editing

**Figure 2:**
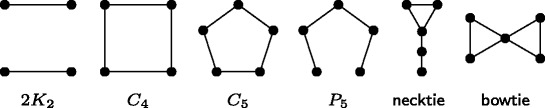
The forbidden induced subgraphs for split graphs (2*K*_2_, *C*_4_, and *C*_5_) and for split cluster graphs (*C*_4_, *C*_5_, *P*_5_, necktie, and bowtie).

Each connected component of the solution has to be a split graph. These graphs can be characterized by forbidden induced subgraphs (see Figure 2).

#### **Lemma1** ([22]).

A graph *G* is a split graph if and only if *G* does not contain an induced subgraph that is a pair of disjoint edges or a cycle of four or five edges, that is, *G* is (2*K*_2_,*C*_4_,*C*_5_)-free.

To obtain a characterization for split cluster graphs, we need to characterize the existence of 2*K*_2_’s within connected components. The following lemma will be useful for this purpose.

#### **Lemma2**.

If a connected graph contains a 2*K*_2_ as induced subgraph, then it contains a 2*K*_2_=(*V*^′^,*E*^′^) such that there is a vertex *v*∉*V*^′^ that is adjacent to at least one vertex of each *K*_2_ of (*V*^′^,*E*^′^).

#### *Proof*.

Let *G* contain the 2*K*_2_{*x*_1_,*x*_2_},{*y*_1_,*y*_2_} as induced subgraph. Without loss of generality, let the shortest path between any *x*_*i*_,*y*_*j*_ be *P*=(*x*_1_=*p*_1_,*p*_2_,…,*p*_*k*_=*y*_1_). Clearly, *k*>2. If *k*=3, then *x*_1_ and *y*_1_ are both adjacent to *p*_2_. Otherwise, if *k*=4, then {*x*_2_,*x*_1_=*p*_1_},{*p*_3_,*p*_4_=*y*_1_} is a 2*K*_2_ and *x*_1_ and *p*_3_ are both adjacent to *p*_2_. Finally, if *k*>4, then *P* contains a *P*_5_ as induced subgraph. The four outer vertices of this *P*_5_ induce a 2*K*_2_ whose *K*_2_’s each contain a neighbor of the middle vertex.

We can now provide a characterization of split cluster graphs (see Figure 2).

#### **Theorem1**.

A graph *G* is a split cluster graph if and only if *G* is a (*C*_4_,*C*_5_,*P*_5_,necktie,bowtie)-free graph.

#### *Proof*.

Let *G* be a split cluster graph, that is, every connected component is a split graph. Clearly, *G* does not contain a *C*_4_ or *C*_5_. If a connected component of *G* contains a *P*_5_, then omitting the middle vertex of the *P*_5_ yields a 2*K*_2_, which contradicts the assumption that the connected component is a split graph. The same argument shows that the graph cannot contain a necktie or bowtie.

Conversely, let *G* be (*C*_4_,*C*_5_,*P*_5_,necktie,bowtie)-free. Clearly, no connected component contains a *C*_4_ or *C*_5_. Assume towards a contradiction that a connected component contains a 2*K*_2_ consisting of the *K*_2_’s {*a*,*b*} and {*c*,*d*}. Then according to Lemma 2 there is a vertex *v* which is, without loss of generality, adjacent to *a* and *c*. If no other edges between the 2*K*_2_ and *v* exist, then {*a*,*b*,*v*,*c*,*d*} is a *P*_5_. Adding exactly one of {*b*,*v*} or {*d*,*v*} creates a necktie, and adding both edges results in a bowtie. No other edges are possible, since there are no edges between {*a*,*b*} and {*c*,*d*}.

This leads to a linear-time algorithm for checking whether a graph is a split cluster graph.

#### **Theorem2**.

There is an algorithm that determines in *O*(*n*+*m*) time whether a graph is a split cluster graph and outputs a forbidden induced subgraph if this is not the case.

#### *Proof*.

For each connected component, we run an algorithm by Heggernes and Kratsch [23] that checks in linear time whether a graph is a split graph, and if not, produces a 2*K*_2_, *C*_4_, or *C*_5_. If the forbidden subgraph is a *C*_4_ or *C*_5_, we are done. If it is a 2*K*_2_, then we find, using the method described in the proof of Lemma 2, in linear time an induced 2*K*_2_ such that there is a vertex *v* that is adjacent to at least one vertex in each *K*_2_. The subgraph induced by this 2*K*_2_ plus *v* is either a *P*_5_, necktie, or bowtie, as shown in the proof of Theorem 1.

In contrast, SPLIT CLUSTER EDITING is NP-hard even in restricted cases. Before proving the hardness, we make the following observation that follows from a simple local improvement argument. It will be used in our hardness proof and also in our algorithms.

#### **Observation1**.

There is an optimal solution to SPLIT CLUSTER EDITING such that

- every degree-one vertex whose neighbor has degree at least two is a periphery vertex, and
- no inserted edge is incident with a periphery vertex.

#### **Theorem3**.

SPLIT CLUSTER EDITING is NP-hard even on graphs with maximum degree 10. Further, it cannot be solved in 2^*o*(*k*)^·*n*^*O*(1)^ or 2^*o*(*n*)^·*n*^*O*(1)^ time if the exponential-time hypothesis (ETH) is true.

#### *Proof*.

We reduce from CLUSTER EDITING:

**Input:** An undirected graph *G*=(*V*,*E*) and an integer *k*.

**Question:** Can *G* be transformed into a cluster graph by applying at most *k* edge modifications?

CLUSTER EDITING is NP-hard [24], even if the maximum degree of the input graph is five [25] and it cannot be solved in 2^*o*(*k*)^·*n*^*O*(1)^ time assuming ETH [25,26].

The reduction works as follows; we assume that the original instance does not contain isolated vertices. Given an instance (*G*,*k*) of CLUSTER EDITING, build a graph *G*^′^=(*V*^′^,*E*^′^) that has the same vertices and edges as *G* and deg*G*(*v*) additional degree-one vertices attached to each *v*∈*V*.

We show that *G* can be transformed by at most *k* edge modifications into a cluster graph if and only if *G*^′^ has a split cluster editing set of size at most *k*. First, if a set *S* of at most *k* edge modifications transforms *G* into a cluster graph $\tilde {G}$, then performing the same modifications on *G*^′^ transforms *G*^′^ into a split cluster graph $\tilde {G'}$: Each connected component of $\tilde {G'}$ contains a clique *K* of $\tilde {G}$ plus deg*G*(*v*) degree-one vertices adjacent to each *v*∈*K*. The set of these degree-one vertices is an independent set.

For the other direction, we show that there is a minimum-cardinality edge modification set *S*^′^ that transforms *G*^′^ into a split cluster graph $\tilde {G}'$, such that performing *S*^′^ on *G* transforms *G* into a cluster graph. By Observation 1 and the fact that each vertex in *G* has degree at least one, we can assume that every vertex in *V*^′^∖*V* is a periphery vertex in $\tilde {G}'$. Consider some vertex *v*∈*V*. If *v* is a periphery vertex in $\tilde {G}'$, then all deg*G*(*v*) edges between *v* and *V*^′^∖*V* are deleted (there are no edges between periphery vertices). Then, however, a solution with the same cost is to delete all deg*G*(*v*) edges between *v* and *V* instead. This solution makes *v* a core vertex with neighbors in *V*^′^ only. Hence, we can assume that *S*^′^ makes every vertex in *V* a core vertex. Since $\tilde {G'}$ is a split cluster graph, each core is a clique and different cores are disjoint. Hence, *S*^′^ transforms *G* into a cluster graph.

This shows the correctness of the reduction. The hardness results follow from the previous hardness results and the fact that the solution size remains the same and that the maximum degree of the constructed graph *G*^′^ is exactly twice the maximum degree of *G*.

This hardness result motivates the study of algorithmic approaches such as fixed-parameter algorithms or ILP formulations. For example, SPLIT CLUSTER EDITING is fixed-parameter tractable for the parameter number of edge modifications *k* by the following search tree algorithm: Check whether the graph contains a forbidden subgraph. If this is the case, branch into the possibilities to destroy this subgraph. In each recursive branch, the number of allowed edge modifications decreases by one. Furthermore, since the largest forbidden subgraph has five vertices, at most ten possibilities for edge insertions or deletions have to be considered to destroy a forbidden subgraph. By Theorem 2, forbidden subgraphs can be found in *O*(*n*+*m*) time. Altogether, this implies the following.

#### **Theorem4**.

SPLIT CLUSTER EDITING can be solved in *O*(10^*k*^·(*n*+*m*)) time.

This result is purely of theoretical interest. With further improvements of the search tree algorithm, practical running times might be achievable.

For example, one could focus on improving the base of the exponential factor by a more elaborate case distinction, either designed manually (e. g. [27]) or automatically [28]. Another approach could be to study parameterized data reduction known as *kernelization* [18,19].

### Monopolar graphs

The class of monopolar graphs is hereditary, and thus it is characterized by forbidden induced subgraphs. The set of minimal forbidden induced subgraphs, however, is infinite [29]; for example among graphs with five or fewer vertices, only the wheel *W*_4_ is forbidden, but there are 11 minimal forbidden subgraphs with six vertices. In contrast to the recognition of split cluster graphs, which is possible in linear time by Theorem 2, deciding whether a graph is monopolar is NP-hard [30]. Algorithmic research is focused on the recognition problem for special graph classes. A fairly general such approach uses a 2-SAT formulation [31,32]. Thus MONOPOLAR EDITING is NP-hard already for *k*=0 edge modifications. As a consequence, it is not fixed-parameter tractable with respect to the number of edge modifications *k* unless P=NP (in contrast to SPLIT CLUSTER EDITING).

## Solution approaches

To evaluate our model, it is helpful to obtain optimal solutions to eliminate or at least estimate the systematic bias that might be introduced by heuristics. We use an integer linear programming (ILP) formulation for this. Since it is not able to solve the hardest instances, we also present a heuristic based on simulated annealing.

### Forbidden subgraph ILP

From Theorem 1, we can easily derive an ILP formulation for SPLIT CLUSTER EDITING. For each (undirected) pair of vertices {*u*,*v*}, we introduce binary variables *e*_*uv*_ indicating whether the edge {*u*,*v*} is present in the solution graph. Defining $\bar e_{\textit {uv}} := 1 - e_{\textit {uv}}$, we have

(1) $$\begin{array}{*{20}l} \text{minimize} & \sum_{{\{u, v\} \in E}} \bar e_{uv} + \sum_{{\{u, v\} \notin E}} e_{uv} \text{ subject to}  \end{array} $$

(2) $$\begin{array}{*{20}l} &\sum_{{\{u, v\} \in E_{F}}} \bar e_{uv} + \sum_{{\{u, v\} \notin E_{F}}} e_{uv} \geq 1 \forall (V_{F}, E_{F}) \in \mathcal{F},  \end{array} $$

where $\mathcal F$ is the set of forbidden induced subgraphs on *V*. A constraint of type (2) forces that at least one edge differs from the forbidden subgraph. Since an *n*-vertex graph may contain *Ω*(*n*^5^) forbidden subgraphs, in practice we use row generation (lazy constraints) and add in a callback only the constraints that are violated; by Theorem 2, we can find a violated constraint in linear time.

The effectivity of ILP solvers is largely based on getting good lower bounds from the LP relaxation. A common technique to improve this further is to add *cutting planes*, that is, inequalities that are already implied by any integral solution, but that cut off part of the polytope of the LP relaxation. We can derive some cutting planes by strengthening the forbidden subgraph constraints. For a *C*_5_, at least two edits are required to obtain a split cluster graph, so we can replace the 1 on the right-hand side by a 2. For a *P*_5_*uvwxy*, we can use

(3) $$\begin{array}{*{20}l} \bar e_{uv} &+ \bar e_{vw} + \bar e_{wx} + \bar e_{xy} + \frac{1}{2} e_{uw} + e_{vx} + \frac{1}{2} e_{wy} + \frac{1}{2} e_{xu} \\&+ \frac{1}{2} e_{yv} \geq 1.  \end{array} $$

A factor $\frac {1}{2}$ is permissible for edits that require at least one more edit; for example inserting {*u*,*w*} produces a necktie. The summand *e*_*uy*_ is omitted, since this insertion produces a *C*_5_, which needs at least two more edits. Similar strengthenings are possible for neckties and bowties.

### Partition variable ILP

Since monopolar graphs have infinitely many forbidden subgraphs, which are NP-hard to find, the forbidden subgraph ILP formulation is not feasible for MONOPOLAR EDITING. We show an alternative formulation based on the observation that if we correctly guess the partition into core and independent set vertices, we can get a simpler forbidden subgraph characterization for both split cluster graphs and monopolar graphs.

#### **Lemma3**.

Let *G*=(*V*,*E*) be a graph and $C \dot {\cup } I = V$ a partition of the vertices. Then *G* is a split cluster graph with core vertices *C* and periphery vertices *I* if and only if *G* does not contain an edge with both endpoints in *I*, nor an induced *P*_3_ with both endpoints in *C*.

#### *Proof*.

“ ⇒”: We show the contraposition. Thus assume that there is an edge with both endpoints in *I* or an induced *P*_3_ with both endpoints in *C*. Then *I* is not an independent set or *C* does not form a clique in each connected component, respectively.

“ ⇐”: We again show the contraposition. If *G* is not a split cluster graph with core vertices *C* and periphery vertices *I*, then it must contain an edge with both endpoints in *I*, or *C*∩*H* does not induce a clique for some connected component *H* of *G*. In the first case we are done; in the second case, there are two vertices *u*,*v*∈*C* in the same connected component with {*u*,*v*}∉*E*. Consider a shortest path (*u*=*p*_1_,…,*p*_*l*_=*v*) from *u* to *v*. If it contains a periphery vertex *p*_*i*_∈*I*, then *p*_*i*−1_,*p*_*i*_,*p*_*i*+1_ forms a forbidden subgraph. Otherwise, *p*_1_,*p*_2_,*p*_3_ is one.

For annotated monopolar graphs, the situation is even simpler. By Definition 3, the two-partition into *C* and *I* exactly demands that *I* is an independent set and *G*[*C*] is a cluster graph or, equivalently, *P*_3_-free.

#### **Lemma4**.

Let *G*=(*V*,*E*) be a graph and $C \dot {\cup } I = V$ a partition of the vertices. Then *G* is a monopolar graph with core vertices *C* and periphery vertices *I* if and only if it does not contain an edge with both endpoints in *I*, nor an induced *P*_3_ whose vertices are contained in *C*.

#### *Proof*.

“ ⇒”: This follows directly from Definition 3.

“ ⇐”: If *G* is not monopolar with core vertices *C* and periphery vertices *I*, then it must contain an edge with both endpoints in *I*, or *G*[*C*] is not a cluster graph. In the first case we are done; in the second case, there is a *P*_3_ with all vertices in *C*, since that is the forbidden induced subgraph for cluster graphs.

From Lemma 3, we can derive an ILP formulation for SPLIT CLUSTER EDITING. As before, we use binary variables *e*_*uv*_ indicating whether the edge {*u*,*v*} is present in the solution graph. In addition, we introduce binary variables *c*_*u*_ indicating whether a vertex *u* is part of the core. Defining $\bar e_{\textit {uv}} := 1 - e_{\textit {uv}}$ and $\bar c_{u} := 1 - c_{u}$, and fixing an arbitrary order on the vertices, we have

(4) $$\begin{array}{*{20}l}  \text{minimize} \sum_{{\{u, v\} \in E}} \bar e_{uv} + \sum_{{\{u, v\} \notin E}} e_{uv} & \text{~subject to}  \end{array} $$

(5) $$\begin{array}{*{20}l} c_{u} + c_{v} + \bar e_{uv} & \geq 1 ~ \forall u, v  \end{array} $$

(6) $$\begin{array}{*{20}l} \bar e_{uv} + \bar e_{vw} + e_{uw} + \bar c_{u} + \bar c_{w} & \geq 1 ~ \forall u \neq v, v \neq w > u.  \end{array} $$

Herein, Constraint (5) forces that the periphery vertices are an independent set and Constraint (6) forces that core vertices in the same connected component form a clique. For MONOPOLAR EDITING, we replace Constraint (6) by

(7) $$\begin{array}{*{20}l} \bar e_{uv} + \bar e_{vw} + e_{uw} + \bar c_{u} + \bar c_{v} + \bar c_{w} \geq 1 ~ \forall u \neq v, v \neq w > u  \end{array} $$

which forces that the graph induced by the core vertices is a cluster graph.

### Data reduction

Data reduction (preprocessing) proved very effective for solving CLUSTER EDITING optimally [8,9]. Indeed, any instance can be reduced to one of at most 2*k* vertices [33,34], where *k* is the number of edge modifications. Unfortunately, the data reduction rules we devised for SPLIT CLUSTER EDITING were not applicable to our real-world test instances. However, Observation 1 allows us to fix the values of some variables of Constraints (4) to (6) in the partition variable ILP for SPLIT CLUSTER EDITING: if a vertex *u* has only one vertex *v* as neighbor and deg(*v*)>1, then set *c*_*u*_=0 and *e*_*uw*_=0 for all *w*≠*v*. Since our instances have many degree-one vertices, this considerably reduces the size of the ILPs.

### Heuristics

The integer linear programming approach is not able to solve the hardest of our instances. Thus, we employ the well-known *simulated annealing* heuristic. This is a local search method, where we try a random modification of our current solution, and accept it if it improves the objective; but to escape local minima, we also accept it with a small probability if it makes the objective worse. More precisely, a change in the objective of *Δ* is accepted with probability exp(−*Δ*/*T*), where the factor *T* is reduced over the course of the algorithm down to zero, such that the algorithm initially explores a larger part of the search space, but eventually settles in a local minimum. We restart the simulated annealing algorithm, where each repetition has a fixed number of steps.

For SPLIT CLUSTER EDITING, we start with a clustering where each vertex is a singleton. As random modification, we move a vertex to a cluster that contains one of its neighbors. Since this allows only a decrease in the number of clusters, we also allow moving a vertex into an empty cluster. For a fixed clustering, the optimal number of modifications can be computed in linear time by counting the edges between clusters and computing for each cluster a solution for SPLIT EDITING in linear time [14]. For MONOPOLAR EDITING, we additionally have a set representing the shared periphery. Accordingly, we allow moving a vertex into another cluster or into the independent set. Here, the optimal number of modifications for a fixed clustering can also be calculated in linear time: all edges in the independent set are deleted, all edges between clusters are deleted, and all missing edges within clusters are added.

## Experimental results

We test exact algorithms and heuristics for SPLIT CLUSTER EDITING (SCE) and MONOPOLAR EDITING (ME) on several PPI networks, and perform a biological evaluation of the modules found. We use three known methods for comparison.

- The algorithm by Luo et al. [3] (“Luo” for short) produces clusters with core and periphery, like SCE, but the clusters may overlap and might not cover the whole graph. Luo produces two types of core–periphery structures, those with a dense core, called *k*-plex core, and those with a star core. In the comparison, we consider only the structures with *k*-plex cores, since this model is closer to our models. For periphery, we consider only neighbors of the core (called 1-periphery by Luo et al. [3]) and not vertices with distance two to the core (called 2-periphery).
- The SCAN algorithm [35], like ME, partitions the graph vertices into “clusters”, which we interpret as cores, and “hubs” and “outliers”, which we interpret as periphery. SCAN is run with several parameter combinations, obtaining different results. For consistency, we select the results where the clusters have the highest modularity, as reported by the SCAN program itself.
- In addition, we compare the solutions of SCE and ME with optimal solutions of Cluster Editing (CE) (see Section ‘Split cluster editing’ for a formal problem definition). The result of such a solution is a cluster graph and the size-1 clusters of this cluster graph are an independent set. Accordingly, we interpret the size-1 clusters as periphery. We solve CE by a simple ILP with row generation, using the characterization by the forbidden subgraph *P*_3_.

### Experimental setup

**Implementation details.** The ILPs and the simulated annealing heuristic were implemented in C++ and compiled with the GNU g++ 4.7.2 compiler. As ILP solver, we used CPLEX 12.6.0. For both formulations, we use the heuristic solution found after 10 rounds as MIP start. For the forbidden subgraph formulation (Section ‘Forbidden subgraph ILP’), in a lazy constraint callback, we find a forbidden subgraph using Theorem 2 and add the corresponding inequality of type (2) to the model. We then delete one of its vertices and try to find another forbidden subgraph, adding up to *n* inequalities per callback.

For the partition variable formulation (Section ‘Partition variable ILP’), we initially add all independent set constraints (5) and those *P*_3_ constraints ((6), (7)) for which the vertices *u*,*v*,*w* induce a *P*_3_ in the input graph. In a lazy constraint callback, we add violated *P*_3_ constraints (usually only a few are needed). These constraints are also used as cutting planes, that is, we already add them in a cutting plane callback when they are violated by the fractional solution. In addition, we use the forbidden subgraphs *C*_4_ and *P*_5_ for SCE and the forbidden subgraph *W*_4_ for ME as cutting planes (Eq. 2). In the cutting plane callbacks, we add the 500 inequalities which are violated the most, if the violation is at least 0.3 (these parameters were heuristically determined).

In the simulated annealing heuristic, we use 20,000 steps and an initial *T*_0_=1, and restart the procedure 100 times.

The test machine is a 4-core 3.6 GHz Intel Xeon E5-1620 (Sandy Bridge-E) with 10 MB L3 cache and 64 GB main memory, running Debian GNU/Linux 7.0. CPLEX was allowed to use up to 8 threads, and we report wall clock times.

**Data.** For comparison of the algorithms, we first use random graphs, where each possible edge is present with probability *p*, to examine variability of running times and limits of feasibility. For more realistic data, we generate subnetworks of the *S. cerevisiae* (yeast) protein interaction network from BioGRID [36]. Our networks contain only physical interactions. For each Gene Ontology (GO) term in the annotations of the *Saccharomyces* Genome Database (SGD) [37], we extract the subnetwork induced by only those proteins that are annotated with this term. We omit networks with fewer than 30 vertices (these can all be solved in less than one second). This yields 178 graphs with up to 2198 vertices, with a median of 66 vertices and 226 edges.

For the biological evaluation, we focus on three particular subnetworks, corresponding to three essential processes: cell cycle, translation, and transcription.^a^ These are important subnetworks known to contain complexes. Table 1 shows some properties of these networks.

**Table 1:** **Input properties of the process networks**

	***n***	***m***	***n***_**lcc**_	***m***_**lcc**_	***C***	***p***	***A***_***C***_	***i***_***g***_
Cell cycle	196	797	192	795	7	148	6.3	1151
Transcription	215	786	198	776	11	54	7.5	1479
Translation	188	2352	186	2351	5	88	27.4	174

Here, *n* is the number of proteins, without singletons, and *m* is the number of interactions; *n*_lcc_ and *m*_lcc_ are the number of proteins and interactions in the largest connected component; *C* is the number of CYC2008 complexes with at least 50% and at least three proteins in the network, *p* is the number of network proteins that do not belong to these complexes, and *A*_C_ is the average complex size. Finally, *i*_g_ is the number of genetic interactions between proteins without physical interaction.

**Biological evaluation.** We evaluate our results using the following measures. First, we examine the coherence of the GO terms in our modules using the semantic similarity score calculated by G-SESAME [38]. We use this score to test the hypothesis that the cores are more stable than the peripheries. If the hypothesis is true, then the GO terms within a core should be more similar than the GO terms in the periphery. Hence, the pairwise similarity score within the core should be higher than in the periphery. We test only terms relating to process, not function, since proteins in the same complex play a role in the same biological process. Since ME, SCAN, and CE return multiple cores and only a single periphery, we assign to each cluster *C* its neighborhood *N*(*C*) as periphery. We consider only clusters with at least two core vertices and one periphery vertex.

Next, we compare the resulting clusters with known protein complexes from the CYC2008 database [39]. Since the networks we analyze are subnetworks of the larger yeast network, we discard for each network the CYC2008 complexes that have less than 50% of their vertices in the current subnetwork, restrict them to proteins contained in the current subnetwork, and then discard those with fewer than three proteins. We test the overlap between the algorithm results and these complexes, treating the complexes as the “ground truth”. We expect that the cores mostly correspond to complexes and that the periphery may contain complex vertices plus further vertices.

### Results

#### Random networks

**Figure 3:**
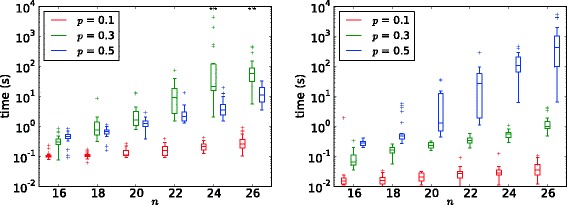
Running times for random graphs. Left: SPLIT CLUSTER EDITING; right: MONOPOLAR EDITING. A star indicates an instance that was aborted due to insufficient memory.

Figure 3 shows running times for random graphs using the fastest ILP version (using partition variables and cutting planes). Each box represents 25 runs. For SCE, running times show large variation (note the logarithmic scale). Density *p*=0.3 here yields harder instances than either denser or sparser instances. Already for *n*=22, two instances with *p*=0.3 could not be solved with available memory, although another one takes only three seconds. For ME and *p*=0.1 or *p*=0.3, there are fewer outliers and the instances can be solved much quicker than for the SCE model. Running times and variance of running time seem to increase monotonously with density, however. Thus, for *p*=0.5SCE could be solved quicker than ME.

The heuristic optimally solves SCE for all instances with known optimal solution; for ME, it is off by one for five instances.

#### PPI subnetworks

**Figure 4:**
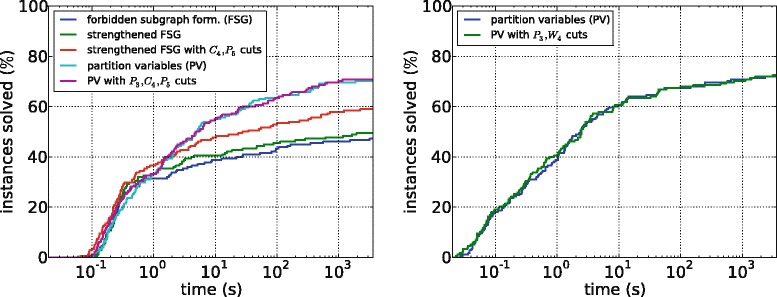
Running times of the different ILP formulations for the PPI subnetworks. Left: SPLIT CLUSTER EDITING; right: MONOPOLAR EDITING.

Figure 4 shows running times for the different ILP approaches on PPI subnetworks. Overall, we can observe that these instances are much easier than the random graph instances. For SCE with the forbidden subgraph formulation, we see that the strengthened inequalities such as Constraint (3) allow to solve more instances, and that using the *P*_5_ (in our instances the most frequent forbidden subgraph) not only as a forbidden subgraph but also as a cutting plane further improves running time. However, neither version is as effective as the partition variable formulation. Here, using forbidden subgraphs as cutting planes has less effect, solving only one more instance. This is probably because adding the initial constraints ((5) to (7)) already produces a fairly tight relaxation. Moreover, finding the cutting planes is quite slow.

For ME, we note that instances can be solved slightly quicker in general, consistent with the observations on sparse random networks. Using *W*_4_ (the smallest forbidden subgraph for monopolar graphs) as a cutting plane also helps little, solving one more instance, but might be useful for difficult instances with long running time.

The heuristic for SCE finds an optimal solution for all 126 instances for which the optimal solution size is known. The ME heuristic optimally solves 104 of 129 instances for which the optimal solution size is known. The average error is very small (0.61), but for one instance the heuristic produces a solution size too high by 27. Possibly the independent set, which interacts with all clusters, makes local search approaches less effective here compared to SCE.

**Figure 5:**
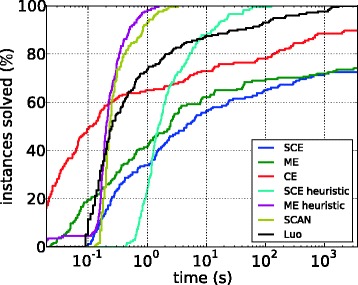
Running times of the best ILP formulations, of the two heuristics, and of LUO and SCAN for the PPI subnetworks.

Figure 5 shows the running times for the fastest ILP approaches, that is, the partition variable ILPs with cuts, and the heuristics for both problems. Also shown are the running times of SCAN, LUO, and the ILP for CE. For the majority of the instances, the ILP approaches for SCE and ME are much slower than all other methods including the ILP for CE. SCAN and the ME heuristic are the fastest methods, solving each instance in less than a minute and most instances within a second. The SCE heuristic is substantially slower than the ME heuristic; this behavior is consistent with the observations for the ILP approaches. Finally, LUO is comparable with the SCE heuristic: it is faster than the exact ILP approaches but substantially slower than SCAN and the ME heuristic.

#### Process networks

Our results are summarized in Table 2 (size statistics and average GO term coherence) and Table 3 (complex detection).

**Table 2:** **Solution statistics and average GO term coherence for the process networks**

	**Cell-cycle**							**Transcription**							**Translation**						
	***k***	***K***	$\bar c$	$\bar p$	***c***_***t***_	***c***_***c***_	***c***_***p***_	***k***	***K***	$\bar c$	$\bar p$	***c***_***t***_	***c***_***c***_	***c***_***p***_	***k***	***K***	$\bar c$	$\bar p$	***c***_***t***_	***c***_***c***_	***c***_***p***_
SCE	321	14	5	7	0.60	0.64	0.40	273	14	6	6	0.54	0.56	0.57	308	6	13	14	0.70	0.73	0.69
ME	126	24	3	9	0.45	0.57	0.40	106	26	3	7	0.50	0.60	0.54	240	11	8	12	0.59	0.61	0.54
SCAN	—	28	5	4	0.41	0.62	0.34	—	29	4	3	0.48	0.59	0.47	—	5	30	4	0.66	0.66	0.76
Luo	—	16	9	63	0.34	0.50	0.31	—	12	8	41	0.40	0.52	0.38	—	4	24	24	0.72	0.84	0.67
CE	461	28	4	1	0.51	0.51	0.38	392	28	4	1	0.56	0.57	0.68	937	10	11	4	0.71	0.73	0.71

Here, *k* is the number of edge modifications, *K* is the number of nontrivial clusters, $\bar {\text {\textit {c}}}$ and $\bar {\text {\textit {p}}}$ are the average size of the core and periphery in a nontrivial cluster, respectively, and *c*_*t*_, *c*_*c*_, and *c*_*p*_ are the average coherence within the cluster, core, and periphery, respectively.

**Table 3:** **Complex detection statistics for the process networks**

	**Cell-cycle**				**Transcription**				**Translation**			
	***D***	**core**_***%***_	**comp**_***%***_	**extra**_***%***_	***D***	**core**_***%***_	**comp**_***%***_	**extra**_***%***_	***D***	**core**_***%***_	**comp**_***%***_	**extra**_***%***_
SCE	4	93/100	100/100	71/ 67	8	87/ 88	100/100	73/ 85	4	89/100	96/ 96	50/ 50
ME	5	89/100	99/100	90/ 89	11	89/100	93/100	76/ 79	4	89/100	95/ 96	55/ 64
SCAN	4	87/ 91	98/100	100/100	8	81/ 84	100/100	96/100	0	—/—	—/—	—/—
Luo	5	76/ 81	100/100	100/100	6	89/ 87	100/100	100/100	4	84/ 92	96/ 96	72/ 73
CE	5	80/ 87	94/100	40/ 0	9	87/ 91	94/100	89/100	4	84/ 92	90/ 94	55/ 60

Here, *D* is the number of detected complexes, core_%_ is among the detected complexes the mean/median percentage of core vertices that are in this complex, comp_%_ is the mean/median percentage of complex proteins that are in the cluster, and extra_%_ is the mean/median percentage of periphery proteins that are not in the cluster.

**Running times and objective function.**

For SCE, the ILP approach failed to solve the cell cycle and transcription network, and for ME, it failed to solve the transcription network, with CPLEX running out of memory in each case. Thus, consistent with the previous types of instances, the theoretically harder ME problem was easier to solve in practice. This could be explained by the fact that the number *k* of necessary modifications is much lower, which could reduce the size of the branch-and-bound tree. For two of the three optimally solved instances, the heuristic finds the optimal solution within one minute. For the third instance (ME transcription) it finds the optimal solution only after several hours; after one minute, it is 2.9% too large. This indicates the heuristic gives good results, and in the following, we use the heuristic solution for the three instances not solvable by ILP. From experiments with other networks, we conjecture that the heuristic SCE solutions are optimal; we are less sure about the heuristic solutions for ME.

As for the PPI subnetworks, the SCAN algorithm runs very fast, finishing within seconds on all three networks; the LUO algorithm is considerably slower as it needs several minutes on the translation network. CE is again slower than LUO but still considerably faster than SCE and ME.

**Cluster statistics and GO term coherence.** Table 2 gives an overview of the number and average sizes of the output clusters and of their average GO term coherence in core and periphery. We say that a cluster is *nontrivial* if it has at least three vertices and at least two core vertices. We describe the results for the cell cycle network in more detail since the results here are the most representative of the three networks. Then, we summarize our findings for the transcription and translation network.

The SCE solution identifies 14 nontrivial clusters; all other clusters are singletons. Only for one of the 14 nontrivial clusters, the GO term coherence is lower in the core than in the periphery (for two clusters the scoring tool does not return a result, four clusters have empty peripheries). This is in line with the hypothesis that cores have higher GO term coherence than peripheries.

The ME result contains more nontrivial clusters than SCE (24). Compared to SCE, clusters have on average about the same size, but a slightly smaller core and a slightly larger periphery (recall that a periphery vertex may occur in more than one cluster). The average coherence in the cores is 0.58, lower than for SCE (0.64), this might be due to the fact that the cores are smaller for ME. On average, coherence in the periphery is much lower than in the cores, but for six clusters it is higher than in the core.

SCAN identifies 7 hubs and 41 outliers, which then comprise the periphery. There are even more nontrivial clusters than for ME. Clusters are smaller than for SCE or ME, in particular the periphery has on average only 4.4 vertices as opposed to 7.3 for SCE or 9.8 for ME. Coherence on cores is similar to SCE and ME, and also lower for the periphery.

LUO outputs only large clusters (this is true for all subnetworks we tested). For the cell cycle network, 16 clusters are identified, each having at least 5 proteins in the cores, and 3 in the periphery, and the largest having 15 proteins in the core and 126 in the periphery (for SCE, one cluster has 10 proteins in the core and 56 in the periphery, and all other clusters for the three other methods have cores of at most 16 and peripheries of at most 30 vertices). The cores have much lower coherence on average than the other methods, but again coherence in the periphery is even lower.

CE outputs many nontrivial clusters, on average the cores and periphery are smaller than for SCE and ME. The average coherence is lower than for SCE and ME, but again the average coherence is higher in the core than in the periphery.

We now describe the results for the transcription network. Again, ME outputs the smallest cores, followed by SCAN and CE. LUO again finds the largest cores and also the largest peripheries. Concerning GO term analysis, we see a similar pattern here that LUO has worse coherence. The average core coherence is the highest for ME and, unlike CE and SCE, the average coherence is higher in the cores than in the periphery for ME.

In the translation network, ME outputs the most nontrivial clusters, followed by CE and SCE. SCAN and LUO output the fewest nontrivial clusters (5 and 4, respectively). LUO has the best coherence values here. The average coherence is higher for CE than for ME but the difference between the average core and periphery coherence is less pronounced in CE than in ME.

**Complex detection.** Table 3 gives an overview of the number of detected complexes. Again, we describe the results for the cell cycle network in more detail and then summarize our findings for the transcription and translation network.

Following our hypothesis, we say that a complex is *detected* by a cluster if at least 50% of the core belongs to the complex and at least 50% of the complex belongs to the cluster. Out of the seven complexes, three are detected without any error (anaphase-promoting, DASH, and Far3p/Far7p/Far8p/Far9p/Far10p/Far11p complex), and one (Mcm2-7) is detected with an error of two additional proteins in the core that are not in the complex. The periphery contains between one and eight extra proteins that are not in the complex (which is allowed by our hypothesis).

ME detects the same complexes as SCE, and additionally the mitotic checkpoint complex. For the anaphase-promoting complex, it misses one protein; all other complexes are detected without error.

SCAN detects almost the same complexes as ME (it misses the Mcm2-7 complex). It also has slightly more errors, for example having three extra protein in the core for the anaphase-promoting complex plus one missing. LUO detects the same complexes as ME without missing any complex proteins but it also finds more extra vertices in the cores. CE detects the same clusters as ME with a slightly higher number of missed complex proteins and extra core proteins.

In the transcription network, the ME method comes out a clear winner: it detects all 11 complexes and has fewer errors than the other methods. CE detects more complexes than SCAN and SCE; LUO detects only 6 complexes for this network.

In the translation network, SCE, ME, LUO, and CE detect the same four complexes. The SCAN algorithm does not seem to deal well with this network, since it does not detect any complex. LUO finds only four nontrivial clusters, corresponding to the four complexes also detected by SCE and ME; this might also explain why it has the best coherence values here.

## Conclusions

### Experiment evaluation

The coherence values for cores and peripheries indicate that a division of clusters into core and periphery makes sense. Under the assumption that cores should be more coherent than peripheries, ME and LUO do best with respect to separating cores from periphery.

In detecting complexes, the ME method does best (20 detected), followed by CE (18), followed by SCE and LUO (15 each), and finally SCAN (12). This indicates that the model that peripheries are shared is superior (note that in CE the size-1 clusters are also a shared periphery). One advantage of ME compared to CE is that the cores are smaller and thus contain fewer extra proteins which are not in the complex. Note that when comparing the number of detected complexes, then SCE is at a disadvantage, since it can use each protein as periphery only once, while having large peripheries makes it easier to count a complex as detected. One approach here could be to consider clusters of size one as shared periphery (as we did for CE). The graph modification-based methods showed a more consistent behavior across the three test networks than LUO (which performs not so well on the transcription network) and SCAN (which performs not so well on the translation network).

A further notable difference between the algorithms is that LUO outputs much larger peripheries for each cluster. Thus, the peripheries of the detected complexes contain many proteins which are not known to be in the complex (by our initial hypothesis, these extra proteins are not necessarily errors). The other four methods are much more conservative in this regard.

### Outlook

Concerning the theoretical analysis of SPLIT CLUSTER EDITING the following questions are open: Is SPLIT CLUSTER EDITING amenable to parameterized data reduction? That is, does SPLIT CLUSTER EDITING admit a polynomial-time reduction to a polynomial-size problem kernel (see [18] for a definition of problem kernel)? Does SPLIT CLUSTER EDITING admit a constant-factor approximation? It would be also interesting to study the SPLIT CLUSTER DELETION problem in which only edge deletions are allowed to transform the input graph into a split cluster graph. This variant is also NP-hard by a reduction that is similar to the one presented for SPLIT CLUSTER EDITING.

For MONOPOLAR EDITING it would be interesting to obtain any tractability results, for example by considering combinations of parameters. A first step here could be to study the problem of recognizing monopolar graphs more closely.

There are many further variants of our models that could possibly yield better biological results or have algorithmic advantages. For instance, one could restrict the cores to have a certain minimum size. Also, instead of using split graphs as a core–periphery model, one could resort to dense split graphs [10] in which every periphery vertex is adjacent to all core vertices. Finally, one could allow some limited amount of interaction between periphery vertices.

Further evaluation of the biological properties of the computed core–periphery structures seems also worthwhile. For example, it would be interesting to examine the peripheries more closely in order to determine whether SPLIT CLUSTER EDITING and MONOPOLAR EDITING are too conservative when determining the periphery of a cluster. Finally, one could explore the biological properties of those clusters that were identified by SPLIT CLUSTER EDITING or MONOPOLAR EDITING but that do not correspond to known protein complexes from the CYC2008 database (all output clusters are listed in the Additional file 1: Supplemental material).

## Endnote

^a^ To determine the protein subsets corresponding to each process, we queried BioMart [40] for all yeast genes annotated with the relevant GO terms: GO:0007049 (cell cycle), GO:0006412 (translation), and GO:0006351 (DNA-templated transcription). Note that this gives somewhat different results than using the SGD GO annotations.

## Additional file

Additional file 1
**Supplemental material.** This file contains the source code of the programs that generate the CPLEX ILPs, our input data (the three process networks), and our output clusters. All files are readable as plain text files.

## References

[1] Spirin V, Mirny LA (2003). Protein complexes and functional modules in molecular networks. PNAS.

[2] Gavin A-C, Aloy P, Grandi P, Krause R, Boesche M, Marzioch M (2006). Proteome survey reveals modularity of the yeast cell machinery. Nature.

[3] Luo F, Li B, Wan X-F, Scheuermann R (2009). Core and periphery structures in protein interaction networks. BMC Bioinformatics.

[4] Leung HC, Xiang Q, Yiu S-M, Chin FY (2009). Predicting protein complexes from PPI data: a core-attachment approach. J Comput Biol.

[5] Wu M, Li X, Kwoh C-K, Ng S-K (2009). A core-attachment based method to detect protein complexes in PPI networks. BMC Bioinformatics.

[6] Ben-Dor A, Shamir R, Yakhini Z (1999). Clustering gene expression patterns. J Comput Biol.

[7] Shamir R, Sharan R, Tsur D (2004). Cluster graph modification problems. Discrete Appl Math.

[8] Böcker S, Briesemeister S, Klau GW (2011). Exact algorithms for cluster editing: Evaluation and experiments. Algorithmica.

[9] Böcker S, Baumbach J (2013). Cluster editing. Proceedings of the 9th conference on computability in Europe (CiE ’13). LNCS.

[10] Borgatti SP, Everett MG (1999). Models of core/periphery structures. Soc Netw.

[11] Chernyak ZA, Chernyak AA (1986). About recognizing (*α*,*β*) classes of polar graphs. Discrete Math.

[12] Della Rossa F, Dercole F, Piccardi C. Profiling core-periphery network structure by random walkers. Sci Rep. 2013. Article no. 3. 10.1038/srep01467 PMC3601366 23507984

[13] Srihari S, Ning K, Leong H (2010). MCL-CAw: a refinement of MCL for detecting yeast complexes from weighted PPI networks by incorporating core-attachment structure. BMC Bioinformatics.

[14] Hammer PL, Simeone B (1981). The splittance of a graph. Combinatorica.

[15] Liu Y, Wang J, Guo J, Chen J (2012). Complexity and parameterized algorithms for cograph editing. Theor Comput Sci.

[16] Hellmuth M, Wieseke N, Lechner M, Lenhof H-P, Middendorf M, Stadler PF (2015). Phylogenomics with paralogs. PNAS.

[17] Zotenko E, Guimarães KS, Jothi R, Przytycka TM. Decomposition of overlapping protein complexes: a graph theoretical method for analyzing static and dynamic protein associations. Algorithms Mol Biol. 2006; 1(7). 10.1186/1748-7188-1-7 PMC1481596 16722537

[18] Downey RG, Fellows MR (2013). Fundamentals of Parameterized Complexity. Texts in Computer Sci.

[19] Niedermeier R (2006). Invitation to fixed-parameter algorithms.

[20] Impagliazzo R, Paturi R, Zane F (2001). Which problems have strongly exponential complexity?. J Comput Syst Sci.

[21] Lokshtanov D, Marx D, Saurabh S (2011). Lower bounds based on the exponential time hypothesis. Bull EATCS.

[22] Foldes S, Hammer PL (1977). Split graphs. Congressus Numerantium.

[23] Heggernes P, Kratsch D (2007). Linear-time certifying recognition algorithms and forbidden induced subgraphs. Nord J Comput.

[24] Křivánek M, Morávek J (1986). NP-hard problems in hierarchical-tree clustering. Acta Informatica.

[25] Fomin FV, Kratsch S, Pilipczuk M, Pilipczuk M, Villanger Y. Subexponential fixed-parameter tractability of cluster editing. CoRR. 2011:abs/1112.4419.

[26] Komusiewicz C, Uhlmann J (2012). Cluster editing with locally bounded modifications. Discrete Appl Math.

[27] Liu Y, Wang J, Xu C, Guo J, Chen J (2015). An effective branching strategy based on structural relationship among multiple forbidden induced subgraphs. J Comb Optimization.

[28] Gramm J, Guo J, Hüffner F, Niedermeier R (2004). Automated generation of search tree algorithms for hard graph modification problems. Algorithmica.

[29] Berger AJ (2001). Minimal forbidden subgraphs of reducible graph properties. Discussiones Mathematicae Graph Theory.

[30] Farrugia A (2004). Vertex-partitioning into fixed additive induced-hereditary properties is NP-hard. Electron J Combinatorics.

[31] Le VB, Nevries R (2014). Complexity and algorithms for recognizing polar and monopolar graphs. Theor Comput Sci.

[32] Churchley R, Huang J (2014). Solving partition problems with colour-bipartitions. Graphs Combinatorics.

[33] Cao Y, Chen J (2012). Cluster editing: Kernelization based on edge cuts. Algorithmica.

[34] Chen J, Meng J (2012). A 2*k* kernel for the cluster editing problem. J Comput Syst Sci.

[35] Xu X, Yuruk N, Feng Z, Schweiger TAJ (2007). SCAN: a structural clustering algorithm for networks. Proceedings of the 13th ACM SIGKDD international conference on Knowledge Discovery and Data mining (KDD ‘07).

[36] Chatr-aryamontri A, Breitkreutz B-J, Heinicke S, Boucher L, Winter AG, Stark C (2013). The BioGRID interaction database: 2013 update. Nucleic Acids Res.

[37] Cherry JM, Hong EL, Amundsen C, Balakrishnan R, Binkley G, Chan ET (2012). Saccharomyces genome database: the genomics resource of budding yeast. Nucleic Acids Res.

[38] Du Z, Li L, Chen C-F, Yu PS, Wang JZ (2009). G-SESAME: web tools for GO-term-based gene similarity analysis and knowledge discovery. Nucleic Acids Res.

[39] Pu S, Wong J, Turner B, Cho E, Wodak SJ (2009). Up-to-date catalogues of yeast protein complexes. Nucleic Acids Res.

[40] Kasprzyk A. BioMart: driving a paradigm change in biological data management. Database. 2011;2011. 10.1093/database/bar049 PMC3215098 22083790

